# Rice miR1432 Fine-Tunes the Balance of Yield and Blast Disease Resistance via Different Modules

**DOI:** 10.1186/s12284-021-00529-1

**Published:** 2021-10-21

**Authors:** Yan Li, Ya-Ping Zheng, Xin-Hui Zhou, Xue-Mei Yang, Xiao-Rong He, Qin Feng, Yong Zhu, Guo-Bang Li, He Wang, Jing-Hao Zhao, Xiao-Hong Hu, Mei Pu, Shi-Xin Zhou, Yun-Peng Ji, Zhi-Xue Zhao, Ji-Wei Zhang, Yan-Yan Huang, Jing Fan, Ling-Li Zhang, Wen-Ming Wang

**Affiliations:** 1 State Key Laboratory of Crop Gene Exploration and Utilization in Southwest China, Sichuan Agricultural University, Chengdu, China; 2 Present Address: College of Environmental Science and Engineering, China West Normal University, Nanchong, China

**Keywords:** miR1432, Blast disease resistance, Yield traits, *OsEFH1*, *OsACOT*

## Abstract

microRNAs act as fine-tuners in the regulation of plant growth and resistance against biotic and abiotic stress. Here we demonstrate that rice miR1432 fine-tunes yield and blast disease resistance via different modules. Overexpression of miR1432 leads to compromised resistance and decreased yield, whereas blocking miR1432 using a target mimic of miR1432 results in enhanced resistance and yield. miR1432 suppresses the expression of *LOC_Os03g59790*, which encodes an EF-hand family protein 1 (*OsEFH1*). Overexpression of *OsEFH1* leads to enhanced rice resistance but decreased grain yield. Further study revealed that miR1432 and *OsEFH1* are differentially responsive to chitin, a fungus-derived pathogen/microbe-associated molecular pattern (PAMP/MAMP). Consistently, blocking miR1432 or overexpression of *OsEFH1* improves chitin-triggered immunity responses. In contrast, overexpression of *ACOT*, another target gene regulating rice yield traits, has no significant effects on rice blast disease resistance. Altogether, these results indicate that miR1432 balances yield and resistance via different target genes, and blocking miR1432 can simultaneously improve yield and resistance.

**Supplementary Information:**

The online version contains supplementary material available at 10.1186/s12284-021-00529-1.

## Background

In plant-pathogen co-evolution, plants employ two-layered immunity to counterattack the invasion of pathogens, namely PAMP/MAMP-triggered immunity (PTI) and effector-triggered immunity (ETI) (Jones and Dangl 2006). PTI is the first layer of plant immunity activated by the recognition of the PAMPs/MAMPs and pattern recognition receptors (PRRs), such as bacterium-derived flg22 and fungus-derived chitin, to effectively protect plants from the invasion of potential pathogens (Boller and Felix 2009). The typical PTI responses include the activities of MAPK cascades, the influx of [Ca^2+^]_cyt_, the burst of reactive oxygen species (ROS), the induction of basal defense-related genes, the callose deposition at the infected sites, and so on (Boller and Felix 2009). However, adapted pathogens can subvert PTI by delivering effectors in host cells (Dou and Zhou 2012). In turn, plants have involved resistance (R) proteins to recognize these specific effectors resulting in ETI, which offers strong resistance and is often associated with the hypersensitive response (HR) (Cui et al. 2015).

microRNAs (miRNAs) are a category of 20–24-nucleotide (nt) non-coding RNAs expressed from *MIR* genes and regulate target gene expression by sequence-complementary DNA methylation or mRNA cleavage, or translational inhibition (Yu et al. 2017). Based on their roles in the regulation of gene expression, miRNAs act as fine-tuners of various biological processes controlling growth and stress-induced responses (Tang and Chu 2017). Growing evidence shows that miRNAs are involved in plant immunity (Padmanabhan et al. 2009; Katiyar-Agarwal and Jin 2010; Baldrich and San Segundo 2016; Huang et al. 2016; Tang and Chu 2017). In Arabidopsis, the PAMP molecule flg22 induces the expression of miR160a and miR393, whereas suppresses the accumulation of other nine miRNAs following the inoculation of the virulent *Pseudomonas syringae* pv. *tomato* DC3000 (*Pst* DC3000) (Navarro et al. 2006; Li et al. 2010). miRNAs are also involved in plant ETI. In Arabidopsis, the amounts of miR863-3p are increased during ETI triggered by *Pseudomonas syringae* carrying effector avrRpt2 (*Pst* DC3000(*avrRpt2*)). miR863-3p fine-tunes the amplitude and timing of defense responses by suppressing the target genes that play reverse functions in rice immunity. At the earlier infection stage, miR863-3p promotes immunity by suppressing the expression of typical receptor-like pseudokinase1 (*ARLPK1*) and *ARLPK2*, which negatively regulate plant defense (Niu et al. 2016). At a later infection stage, miR863-3p limits immunity amplitude by silencing *SERRATE*, which is required for miRNA accumulation and positively regulates plant defense (Niu et al. 2016).

Rice blast disease caused by *Magnaporthe oryzae* (*M. oryzae*) ranks the first fungal disease threatening food production worldwide. The utilization of disease resistance genes in cultivars generates an economically and environment-friendly strategy for disease control. Intriguingly, miRNAs play important roles in rice resistance against *M. oryzae* (Li et al. 2014, 2016). Nowadays, more than 15 miRNAs have been characterized as the regulators of rice blast disease resistance. miR159 (Chen et al. 2021), miR160 (Li et al. 2014), miR162 (Salvador-Guirao et al. 2019; Li et al. 2020), miR166 (Salvador-Guirao et al. 2018), miR398 (Li et al. 2019), miR7695 (Campo et al. 2013), and miR812w (Campo et al. 2021) positively regulate rice resistance against *M. oryzae*, whereas miR156 (Zhang et al. 2020), miR164 (Wang et al. 2018b), miR167 (Zhao et al. 2019b), miR169 (Li et al. 2017), miR319 (Zhang et al. 2018), miR396 (Chandran et al. 2019), miR439 (Lu et al. 2021), miR444b.2 (Xiao et al. 2017), and miR1873 (Zhou et al. 2020) negatively regulate rice disease resistance. Among these miRNAs, some and their target genes are involved in both rice immunity and growth. For example, miR162 balances rice resistance and grain yield via *Dicer-like 1* (*OsDCL1*). Overexpression of miR162 enhances rice blast resistance whereas compromises yield accompanied by the suppressed expression of *OsDCL1*; in contrast, blocking miR162a improves yield whereas penalizes immunity associated with enhanced expression of *OsDCL1* (Salvador-Guirao et al. 2019; Li et al. 2020).

miR1432 is a conserved miRNA family in plants involving in development and defense responses against biotic or abiotic stresses. In barley, the amounts of miR1432-5p are increased during barley development (Pacak et al. 2016). In maize, miR1432 is down-regulated in meristem under chilling stress (Aydinoglu 2020). In wheat, miR1432 in leaves is down-regulated by water deficit in presence of mycorrhizal treatment (Fileccia et al. 2019). In wild emmer wheat (*Triticum turgidum* ssp. *dicoccoides*), the expression of miR1432 is induced in roots under drought stress (Kantar et al. 2011). In rice, miR1432 is predicted as a key regulator to regulates rice grain-filling and targets *Acyl-CoA thioesterase* (*OsACOT*) (Hu et al. 2018). Blocking miR1432 significantly enhances grain weight resulting in overall grain yield up more than 17% via expressing a Short Tandem Target Mimic of miR1432 (STTM1432), and overexpression of *OsACOT* resembled the yield traits of STTM1432 plants (Zhao et al. 2019a), indicating miR1432 is involved in grain-filling via *OsACOT*. Except for the regulation of grain yield, miR1432 is also responsive to the infection of *M. oryzae* (Li et al. 2014), indicating the involvement of rice blast disease resistance. However, it remains largely unknown whether miR1432 controls rice resistance and coordinates resistance and yield traits.

In this study, we constructed the transgenic lines overexpressing miR1432, the lines blocking miR1432 by expressing a target mimic of miR1432, and the lines overexpressing the target genes of miR1432, *OsACOT*, and *OsEFH1* (EF-hand family protein 1), respectively. We explored the blast disease resistance and yield traits of these lines. We found that blocking miR1432 leads to enhanced blast disease resistance and increased yield. We revealed that *OsEFH1* was targeted by miR1432 and acted as a positive regulator of rice blast disease resistance but a negative regulator of rice yield. Further study revealed that the miR1432-*OsEFH1* module regulated rice PTI responses, whereas *OsACOT* had no obvious effect on rice resistance. Our results revealed that a miRNA coordinates rice yield and immunity via different target genes that play differential roles, and indicated the capacity of the miR1432-targets module in the improvement of both immunity and yield in rice.

## Results

### Overexpression of miR1432 Compromises Rice Blast Disease Resistance

In rice, one *MIR1432* gene was identified locating on chromosome 7. We examined the expression pattern of miR1432 upon *M. oryzae* treatment in a susceptible rice accession Lijiangxin Tuan Heigu (LTH) and a resistant accession International Rice Blast Line Pyricularia-Kanto51-m-Tsuyuake (IRBLkm-Ts). LTH is a *japonica* accession highly sensitive to over 1300 isolates of *M. oryzae* worldwide, and no major *R* genes are ever identified in it (Lin et al. 2001). IRBLkm-Ts contains an *R* gene locus *Pikm* that mediates ETI against *M. oryzae* strains expressing the avirulence gene alleles *AVR-PikA*/*D*/*E* (Tsumematsu et al. 2000; Kanzaki et al. 2012). LTH exhibited serious disease lesions following the spray-inoculation of Guy11 (Additional file 1: Figure S1a). In contrast, IRBLkm-Ts only showed a few and small resistance lesions (Additional file 1: Figure S1a). The amounts of miR1432 in LTH were unchanged at 12 h post-inoculation (hpi) of *M. oryzae* but decreased at 24 hpi, then significantly increased at 48 hpi. Different from that in LTH, miR1432 slightly fluctuated in IRBLkm-Ts (Additional file 1: Figure S1b). These results indicated that miR1432 is responsive to *M. oryzae*.

**Fig. 1:**
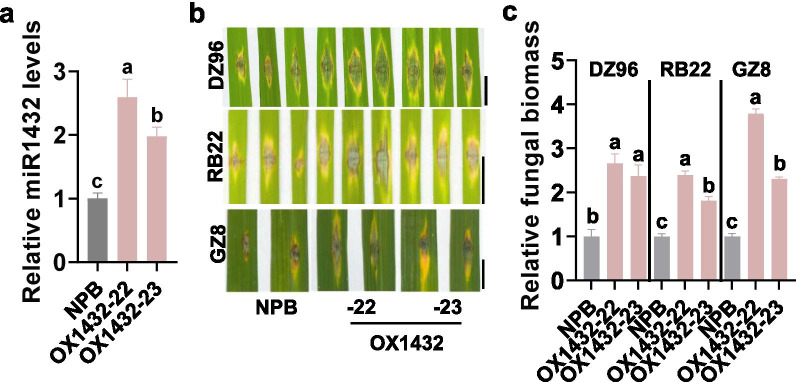
Overexpression of miR1432 enhances rice susceptibility to *Magnaporthe oryzae*. **a** The relative amount of mature miR1432 in the transgenic lines overexpressing *MIR1432* gene (OX1432) and the Nipponbare (NPB) control. Reverse-transcription (RT) was carried out with total RNA and a miR1432 specific stem-loop RT primer (Additional file 2: Table S2). The RT product was subsequently used as a template for quantitative polymerase chain reaction (q-PCR) to detect the amounts of miR1432. The amounts of snRNA U6 were examined and used as an internal reference. **b** The blast disease phenotypes on leaves 5 days post-inoculation of *M. oryzae* strains GZ8, RB22, and DZ96, respectively. Bar = 5 mm. **c** The relative fungal biomass of the indicated strains in OX1432 and the Nipponbare control. The fungal biomass was determined by using the ratio of DNA levels of *M. oryzae MoPot2* against the DNA levels of rice *ubiquitin*. Error bars indicate SD (n = 3 independent samples). Different letters above the bars indicate significant differences (*P* < 0.01) as determined by One-way Tukey–Kramer analysis. Similar results were obtained in at least two independent experiments

To investigate the roles of miR1432 in rice immunity, we constructed the transgenic lines overexpressing *MIR1432* (OX1432). We got more than 25 transgenic lines and selected two lines, namely OX1432-22 and OX1432-23, for further study. The two OX1432 lines showed significantly higher miR1432 accumulation than the Nipponbare (NPB) control (Fig. 1a). We selected three *M*. *oryzae* strains for disease assays. DZ96 is a strain derived from a paddy yard in the Sichuan Basin, Southwest China. RB22 is derived from a paddy yard in North China. GZ8 is a GFP-tagged strain Zhong8-10-14 derived from North China. OX1432 lines were more susceptible to these strains with significantly larger disease lesions and supported more fungal biomass by punch-inoculation (Fig. 1b–d). These results indicate that miR1432 compromises rice resistance against *M. oryzae*.

### Blocking miR1432 Results in Enhanced Rice Blast Disease Resistance

**Fig. 2:**
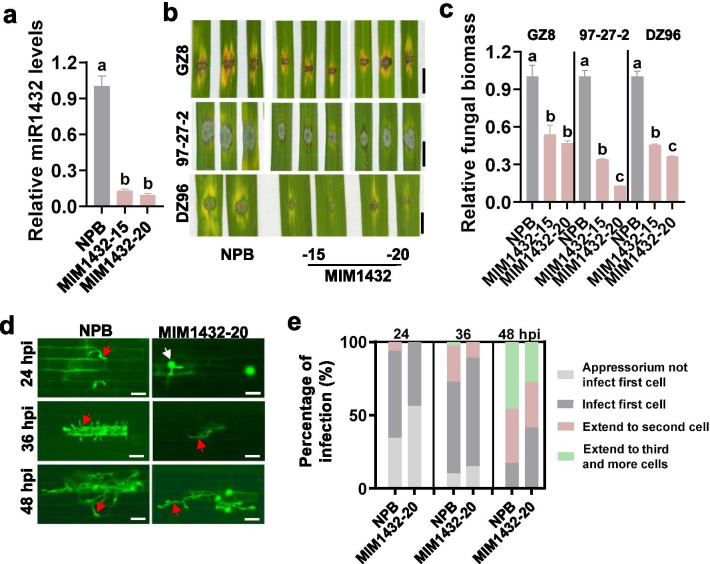
Expression of a target mimic of miR1432 (MIM1432) enhances rice resistance against *Magnaporthe oryzae*. **a** The relative amounts of miR1432 in MIM1432 and the Nipponbare (NPB) control. Reverse-transcription (RT) was conducted with total RNA and a miR1432 specific stem-loop RT primer (Additional file 2: Table S2). The RT product was used as a template for quantitative polymerase chain reaction (q-PCR) to examine the amounts of miR1432. snRNA U6 was served as an internal reference. **b** The blast disease phenotypes on leaves of MIM1432 and the Nipponbare control at 5 days post-inoculation of *M. oryzae* strains GZ8, 97-27-2, and DZ96. Bar = 5 mm. **c** The relative fungal biomass of the indicated strains on MIM1432 and the Nipponbare control in (b). The relative fungal biomass was determined by using the ratio of DNA levels of the *M. oryzae Pot2* against the DNA levels of rice *Ubiquitin*. **d** The invasion process of GZ8 at 24, 36, and 48 h post-inoculation (hpi) in sheath cells of the indicated lines. Bars = 40 µm. The white arrows indicate appressoria formed from conidia, and the red arrowheads indicate invasive hypha in rice sheath cells. **e** Quantification analysis of the fungal development during the invasive process. Over 200 conidia in each line were analyzed. For **a** and **c**, error bars indicate SD (n = 3 independent samples). Different letters above the bars indicate significant differences (*P* < 0.01) as determined by One-way Tukey–Kramer analysis. Similar results were obtained in at least two independent experiments

To further investigate the roles of miR1432 in rice immunity, we made transgenic lines expressing a target mimic of miR1432 (MIM1432). MIM1432 blocks miR1432 from suppressing its target genes by forming a double-strand complex with miR1432 (Additional file 1: Figure S2). MIM1432 lines showed significantly lower miR1432 accumulation than the Nipponbare control (Fig. 2a). We also selected three *M*. *oryzae* strains for disease assays. 97-27-2 is a virulence strain derived from a paddy yard in North China. MIM1432 exhibited enhanced resistance against the three strains with smaller disease lesions and supported less fungal biomass than that of the Nipponbare control (Fig. 2b, c). Moreover, when inoculated with GZ8, MIM1432 displayed delayed infection progress. At 36 hpi, the invasive hyphae filled the whole primary cells in the Nipponbare control whereas just invaded into the primary cells in MIM1432 (Fig. 2d). At 48 hpi, the invasive hyphae were vigorously grown in the Nipponbare control, whereas only fewer hyphae grew into the second cell in MIM1432 (Fig. 2d). Quantification analysis revealed that GZ8 became less aggressive in MIM1432 than in the Nipponbare control (Fig. 2e). We also conducted disease assays on MIM1432 by spray-inoculation of the virulence strain RB22. MIM1432 exhibited fewer and smaller disease lesions than the Nipponbare control (Additional file 1: Figure S3a). Consistent with the disease phenotype, MIM1432 supported less fungal growth than the Nipponbare control (Additional file 1: Figure S3b). In a summary, blocking miR1432 enhances rice blast resistance and delays the colonization of *M. oryzae*.

Then, we examined the expression of defense-related genes in OX1432 and MIM1432 following inoculation of RB22. RB22 induced the expression of the defense-related genes, namely *NAC DOMAIN-CONTAINING PROTEIN 4* (*NAC4*), *ENT-KAURENE SYNTHASE 4* (KS4), and *PATHOGENESIS-RELATED GENE 1a* (*PR1a*) (Li et al. 2020). The expression of these genes was constitutively higher in MIM1432 than that in the Nipponbare control, whereas was unchanged in OX1432 (Additional file 1: Figure S3c), indicating that blocking miR1432 enhanced rice defense responses to fight against *M. oryzae*.

### *OsEFH1* is Targeted by miR1432

**Fig. 3:**
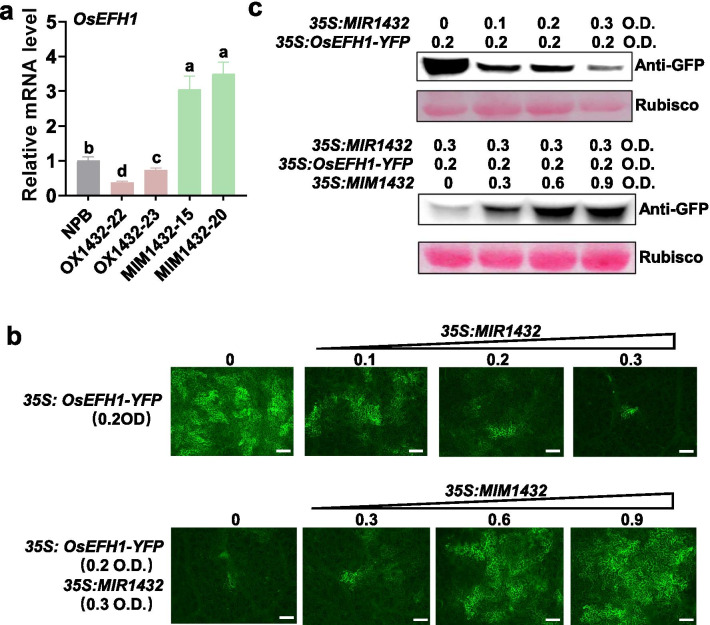
miR1432 suppresses the expression of *OsEFH1*. **a** The relative mRNA levels of *OsEFH1* in OX1432, MIM1432, and the Nipponbare (NPB) control. Error bars indicate SD (n = 3 independent samples). Different letters above the bars indicate significant differences (*P* < 0.01) as determined by One-way Tukey–Kramer analysis. **b** The YFP intensity of OsEFH1-YFP fused protein in *Nicotiana benthamiana* leaves. The *OsEFH1-YFP* constructs were transiently expressed alone or co-expressed with miR1432 alone or co-expressed with miR1432 and MIM1432 together in *Nicotiana benthamiana* leaves using Agrobacterium-mediated infiltration at the indicated concentration (Optical Density (O. D.)). Bar = 100 μm. **c** Western blotting assay indicates the protein levels of OsEFH1-YFP in (B). All the experiments were repeated two times with similar results

We next explored how blocking miR1432 improved rice immunity. miRNAs regulate plant development and defense responses via their target genes. *LOC_Os03g59790* is predicted as a target gene of miR1432 in rice (https://www.zhaolab.org/psRNATarget/) and encodes an EF-hand family protein (http://rice.plantbiology.msu.edu). We named it *OsEFH1* (Additional file 1: Figure S2). The mRNA levels of *OsEFH1* were decreased in OX1432 whereas increased in MIM1432 in comparison with that in the Nipponbare control (Fig. 3a), and were inversely correlated with the expression of miR1432 in these lines (Figs. 1a, 2a), suggesting the suppression by miR1432. To confirm the suppression of miR1432 on *OsEFH1*, we made constructs expressing yellow fluorescence protein (YFP)-fused *OsEFH1* (*35S: OsEFH1-YFP*). The YFP intensity and protein level expressed from *35S: OsEFH1-YFP* were decreased following the co-expression of miR1432 in *N. benthamiana*, but recovered when MIM1432 was co-expressed (Fig. 3b, c). These results indicate that miR1432 represses the expression of *OsEFH1*.

### *OsEFH1* Positively Regulates Rice Resistance Against *M. oryzae*

**Fig. 4:**
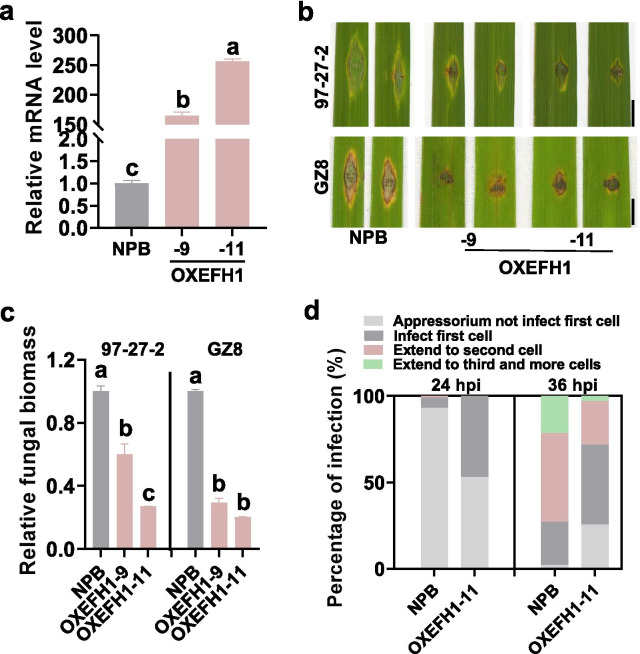
*OsEFH1* enhances rice blast disease resistance. **a** The relative mRNA levels of *OsEFH1* in transgenic lines overexpressing *OsEFH1* (OXEFH1) and the Nipponbare (NPB) control. Total RNA was extracted from three to five-leaf stage seedlings for reverse-transcription-quantitative polymerase chain reaction (RT-qPCR) analysis. The relative mRNA levels of *OXEFH1* lines were normalized with that of the Nipponbare control. **b** The blast disease phenotypes of OXEFH1 lines and the Nipponbare control at 5 days post-inoculation (dpi) of indicated *M. oryzae* strains. Bar = 5 mm. **c** The Quantification analysis of the relative fungal biomass in **b**. The fungal biomass was shown as the ratio of DNA level of *M. oryzae MoPot2* genes against that of rice *ubiquitin*. **d** Quantification analysis of the fungal development during the invasive process. Over 200 conidia in each line were analyzed. For **a** and **c**, error bars indicate SD (n = 3 independent samples). Different letters above the bars indicate a significant difference (*P* < 0.01) as determined by a one-way Tukey–Kramer analysis. All the experiments were repeated two times with similar results

We first examined the expression pattern of *OsEFH1* following *M. oryzae* inoculation. The treatment of *M. oryzae* enhanced the expression of *OsEFH1* in both LTH and IRBLkm-Ts (Additional file 1: Figure S4a), suggesting the participation in rice blast disease resistance. To further explore the role of *OsEFH1* in rice immunity, we constructed the transgenic lines overexpressing *OsEFH1* (OXEFH1). We selected two transgenic lines displaying a significantly higher expression of *OsEFH1* for disease assay (Fig. 4a). OXEFH1 showed enhanced resistance with smaller disease lesions and supported less fungal growth than the Nipponbare control by punch-inoculation or spray-inoculation of *M. oryzae* strains (Fig. 4b, c; Additional file 1: Figure S4b, c). When infected by the virulence strain GZ8, OXEFH1 displayed the delayed infection progress of GZ8 in comparison with that of the Nipponbare control (Fig. 4d; Additional file 1: Figure S4d). Moreover, GZ8 induced more H_2_O_2_ accumulation in OXEFH1 than that in the Nipponbare control at 48 hpi (Additional file 1: Figure S4d). These results indicate that *OsEFH1* improves rice blast disease resistance and defense responses.

### miR1432-*OsEFH1* Module Regulates Rice PTI Responses

**Fig. 5:**
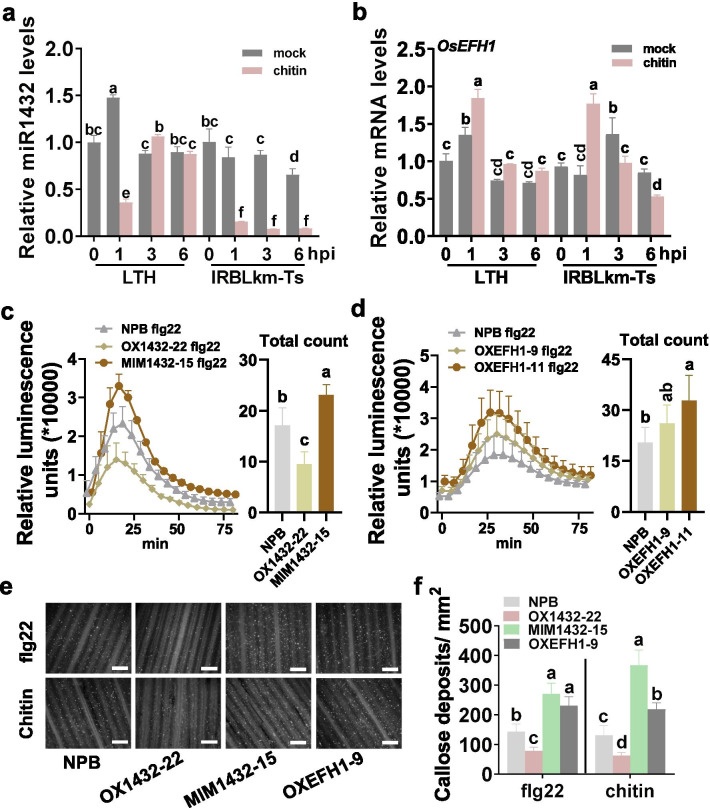
miR1432-*OsEFH1* module regulates rice PTI responses. **a**, **b** The reverse transcription-quantitative polymerase chain reaction (RT-qPCR) data show the amounts of miR1432 (a) and the mRNA levels of *OsEFH1* (b) in LTH and IRBLkm-Ts with or without chitin treatment. Data are shown as mean ± SD (n = 3 independent samples). **c**, **d** The burst of reactive oxidative species (ROS) induced by flg22 in the leaves of indicated lines and the Nipponbare control, respectively. Data are shown as mean ± SD (n = 4 independent repeats). The left diagrams show the relative ROS levels at each timepoint, the right diagrams show the total ROS count. **e** PAMPs (flg22 and chitin) induced callose deposition in the leaves of indicated lines and the Nipponbare control. Bar = 0.5 mm. **f** Quantitative analysis of PAMPs-induced callose deposition in **e**. Data are shown as mean ± SD (n = 6 independent repeats). For a, b, c, d, and f, different letters above the bars show significant differences (*P* < 0.01) as determined by the One-way Tukey–Kramer test. These experiments were repeated two times with similar results

EFH proteins are a sort of proteins binding [Ca^2+^]_cyt_, the influx of which is an important PTI response in cells (Dodd et al. 2010; Boudsocq et al. 2010) and required for a series of responses downstream such as ROS burst (Ranf et al. 2011). We next tested whether miR1432 and *OsEFH1* were responsive to chitin, a well-known fungus-derived PAMP that could induce PTI responses in rice. The expression of miR1432 and *OsEFH1* was enhanced or suppressed in the chitin-treated samples in comparison with the mock sample, demonstrating the regulation by chitin. The amounts of miR1432 in LTH and IRBLkm-Ts were decreased at one hpi of chitin, whereas the expression of *OsEFH1* was increased (Fig. 5a, b), indicating that chitin could trigger the expression of *OsEFH1* via suppressing miR1432, and the miR1432-*OsEFH1* module was possibly involved in PTI. However, the expression pattern of miR1432 was not consistent in LTH and IRBLkm-Ts, suggesting the existence of other regulation pathway in the two accessions. Different form LTH, IRBLkm-Ts contains the *R* gens, namely *Pikm1-Ts* and *Pikm2-Ts*, which cooperatively conferred *Pikm*-specific resistance (Ashikawa et al. 2008). Further study revealed that chitin regulated the expression of the two *R* genes in IRBLkm-Ts (Additional file 1: Figure S5). Whether the *R* genes affected the accumulation of miR1432 is unknown and need further study.

We then examined PAMP-triggered defense responses in the transgenic lines, including ROS burst and callose deposition. MIM1432 and OXEFH1 exhibited higher, whereas OX1432 displayed a lower burst of ROS than the Nipponbare control following the treatment of flg22, a well-known bacteria-derived PAMP (Fig. 5c, d). However, we were failed in the detection of chitin-induced ROS in rice. We examined flg22- and chitin-induced ROS by transient expressing *MIR1432* with or without *MIM1432* or *OsEFH1* in *N. benthamiana* (Additional file 1: Figure S6a–c). The miR1432 was accumulated in the leaves transiently expressing *MIR1432* whereas decreased following the co-expression of MIM1432 (Additional file 1: Figure S6a). The ROS levels were decreased in the leaves transiently expressing *MIR1432* alone in comparison with that in the control leaves transiently expressing *YFP*, whereas were recovered in the leaves co-expressing *MIM1432* or *OsEFH1*, respectively (Additional file 1: Figure S6d–e). Moreover, the MIM1432 and OXEFH1 transgenic lines exhibited more, whereas OX1432 displayed fewer callose deposits triggered by flg22 and chitin than the Nipponbare control (Fig. 5e, f). Altogether, these results demonstrated that the miR1432 negatively regulated PTI responses via suppressing the expression of *OsEFH1*. While overexpression of miR1432 compromises PTI responses, blocking miR1432 or overexpression of *OsEFH1* improves PTI responses.

### *OsACOT* is Not Involved in miR1432-Regulated Rice Blast Disease Resistance

**Fig. 6:**
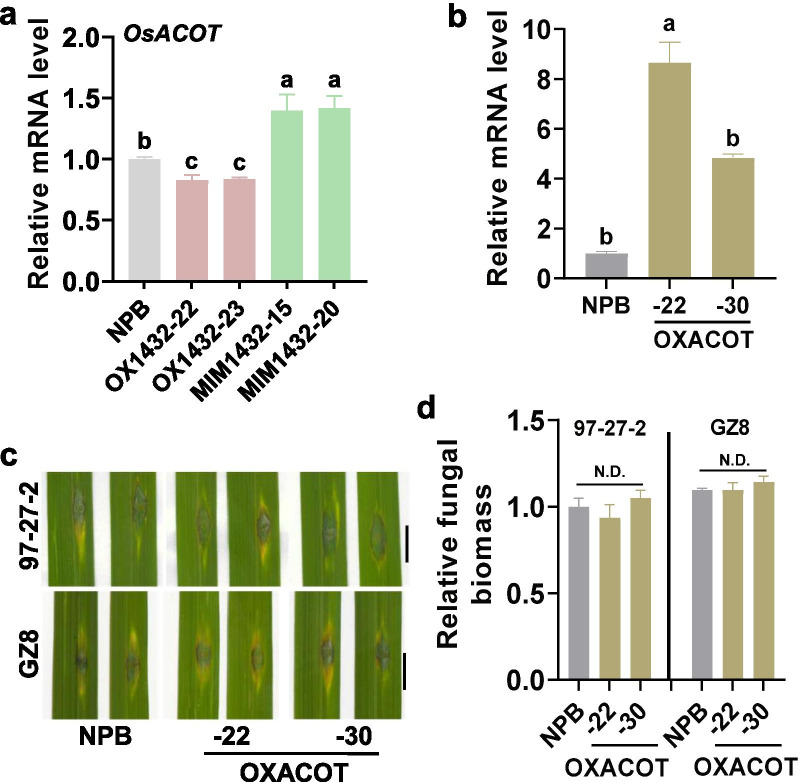
*OsACOT* is not involved in the regulation of rice blast resistance. **a** The relative mRNA levels of *OsACOT* in OX1432, MIM1432, and the Nipponbare control (NPB). **b** The relative mRNA levels of *OsACOT* in the transgenic lines overexpressing *OsACOT* (OXACOT) and the Nipponbare control. For a and b, total RNA was extracted from three to five-leaf stage seedlings for reverse transcription-quantitative polymerase chain reaction (RT-qPCR) analysis. **c** Blast disease phenotypes of OXACOT and the Nipponbare control at 5 days post-inoculation (dpi) of indicated *M. oryzae* strains. Bar = 5 mm. **d** Quantification analysis of the relative fungal biomass in c. The relative fungal biomass was measured by using the ratio of DNA level of *M. oryzae MoPot2* genes against that of rice ubiquitin. For a, b, and d, error bars indicate SD (n = 3 independent samples). N.D. indicates no difference as determined by a one-way ANOVA analysis. All the experiments were repeated two times with similar results

Except for *OsEFH1*, *LOC_Os04g35590* was identified as another target of miR1432 and encoded an Acyl-CoA thioesterase (*OsACOT*) in rice (Zhao et al. 2019a). Overexpression of *OsACOT* enhanced rice yield by improving grain size (Zhao et al. 2019a). The expression of *OsACOT* was suppressed in OX1432 whereas enhanced in MIM1432 in comparison with that in the Nipponbare control (Fig. 6a). We then constructed the transgenic lines overexpressing *OsACOT* (OXACOT), which exhibited significantly higher mRNA levels of *OsACOT* (Fig. 6b). We explored the resistance of OXACOT and found that OXACOT lines displayed the unchanged resistance with similar disease lesions and relative fungal biomass in comparison with the Nipponbare control (Fig. 6c, d). These results indicate that *OsACOT* is not involved in miR1432-regulated blast disease resistance.

### miR1432-Target Modules Regulate Rice Yield Traits

**Fig. 7:**
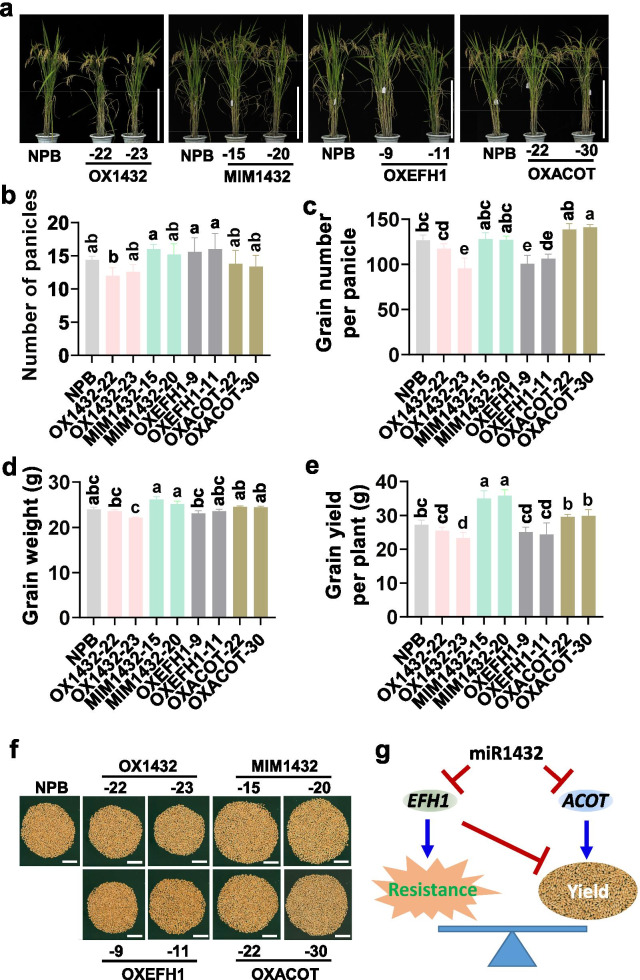
miR1432 and its target genes regulate rice yield traits. **a** The gross morphology of the OX1432, MIM1432, OXEFH1, and OXACOT lines planted in a paddy yard during the normal growing season in Sichuan province, the Southwest of China in 2020. Scale bars, 50 cm. **b**–**e** the panicle number, grain number per panicle, grain weight, and grain yield per plant of the indicated lines. Data are shown as mean ± SD (n = 5 independent samples). Different letters above the bars show significant differences (*P* < 0.05) as determined by the One-way Tukey–Kramer test. **f** Photo of grains per plant of the OX1432, MIM1432, OXEFH1, OXACOT lines, and the Nipponbare control. Bars = 5 cm. **g** A model of miR1432 coordinates rice immunity and grain yield via *OsEFH1* and *OsACOT1* that play different roles in the regulation of yield and resistance

Except for the regulation of immunity, miR1432 and its target genes also control rice agronomical traits. Blocking miR1432 or overexpressing miR1432-insensitive *OsACOT* significantly boosted grain weight leading to increased grain yield (Zhao et al. 2019a). In this study, we examined the yield traits of OX1432, MIM1432, OXEFH1, and OXACOT. We observed pleiotropic phenotypes in OX1432 and MIM1432 planted in a paddy field during the normal growing season from 2018 to 2020, and OXEFH1 and OXACOT lines in 2020. Rice grain yield was determined by three components, including panicle number, panicle size (relying on grain number per panicle and seed setting rate (SSR)), and grain size. All the transgenic lines exhibited a normal plant architecture except that OX1432 showed significantly shorter plants than the Nipponbare control (Fig. 7a, Additional file 2: Table S1). The yield traits of OX1432, MIM1432, and OXACOT in our study were consistent with the phenotype of the transgenic lines in a previous report (Zhao et al. 2019a). OX1432 displayed slightly decreased panicle number, grain number per panicle, and grain weight resulting in decreased yield per plant with a 2.1 to 23.5% reduction in normal rice-growing season from 2018 to 2020 than the Nipponbare control (Fig. 7b–f; Additional file 2: Table S1). Conversely, MIM1432 showed more panicles and grains per panicle, as well as heaver grains resulting in a 2.1% to 31.5% increased yield in three years (Fig. 7b–f; Additional file 2: Table S1). OXEFH1 displayed decreased grain number leading to a 8.0 to 10.6% reduction, whereas OXACOT showed increased grain number and grain weight leading to a 8.7 to 9.2% increase (Fig. 7b–f; Additional file 2: Table S1). These results indicate that miR1432 controls rice yield by suppressing the expression of different target genes that play negative and positive roles in the regulation of rice yield traits. Altogether, miR1432 coordinates blast disease resistance and yield via different target genes that playing different roles in rice immunity and yield traits.

## Conclusions

Altogether, our results reveal that miR1432 fine-tunes rice resistance and yield via different target genes. Overexpression of miR1432 results in decreased rice blast disease resistance accompanied by compromised PTI responses and reduced yield, whereas blocking miR1432 leads to enhanced resistance associated with enhanced PTI responses and increased yield. Further study reveals that *OsEFH1* positively regulates resistance and PTI responses, whereas negatively regulates yield; in contrast, *OsACOT* has no significant effect on rice resistance but positively regulates yield traits. Thus, miR1432 regulates rice yield and resistance via different target genes, and the miR1432-target modules can be used to coordinate resistance and yield in rice production.

## Discussion

In this study, the chitin-induced expression patterns of miR1432 and *OsEFH1* are different in LTH and IRBLkm-Ts. miR1432 accumulation was suppressed by chitin at one hpi and recovered subsequently in the susceptible accession LTH, whereas was suppressed at all the three timepoints in the resistance accession IRBLkm-Ts (Fig. 5a, b), indicating that chitin triggered unknown mechanism to suppress the accumulation of miR1432 thus improved the chitin-triggered resistance in IRBLkm-Ts (Fig. 5c–f). In IRBLkm-Ts, the *Pikm* genes were responsive to chitin, the treatment of which enhanced the transcription of *Pikm2-Ts* at one and three hpi (Additional file 1: Figure S5), indicating that chitin possibly could trigger *Pikm*-mediated resistance. These results were consistent with the recent reports showing that PTI and ETI was united and the PTI receptors were required for NLR-mediated resistance (Yuan et al. 2021; Ngou et al. 2021). In turn, the product of *Pikm* genes possibly affected the chitin-triggered transcriptional reprogramming in IRBLkm-Ts, including the transcription of miR1432 and *OsEFH1*. Thus, chitin triggered different expression pattern in LTH and IRBLkm-Ts, and the lasting-reduced miR1432 in IRBLkm-Ts is helpful to enhance PTI-related defense responses in IRBLkm-Ts.

In this study, the expression of *OsEFH1* was not correlated with that of miR1432 in LTH and IRBLkm-Ts. miR1432 was significantly down-regulated after chitin treatment (3-6hpi), but *OsEFH1* was not up-regulated In IRBLkm-Ts (Fig. 5a, b). It is well known that the expression of genes is regulated at transcriptional level, post-transcriptional level, and translational level. The transcriptional regulation is the major and key regulation, whereas the miRNAs-mediated post-transcriptional and translational regulation play minor roles and fine-tune gene expression (Tang and Chu 2017). Consistently, *OsEFH1* should be regulated at transcriptional level first, then fine-tuned by miR1432. As a result, the expression pattern of *OsEFH1* was inconsistent with the expression pattern of miR1432 (Fig. 5a, b).

Plant immunity often restricts yield in crops (Nelson et al. 2018). In a review of disease resistance studies, 56% of the studies reported the trade-offs between resistance and biomass or fecundity (Bergelson and Purrington 1996). However, increasing evidence reveals that resistance and yield can be simultaneously achieved. For example, fitness costless broad-spectrum disease resistance can be engineered via expression of the Arabidopsis *Non-expressor of Pathogenesis-Related genes1* (*NPR1*) in rice controlled by pathogen-inducible upstream open reading frame (uORF) (Xu et al. 2017). The *Pigm*/*Pi50* locus mediates fitness costless rice blast resistance via an epigenetic regulation (Deng et al. 2017; Su et al. 2015). The transcription factor Ideal Plant Architecture 1 (IPA1) promotes both yield and immunity via phosphorylation-mediated switching of binding specificity to the promoter of *Dense and Erect Panicle 1*(*DEP1*) and *WRKY45* with or without infection of *M*. *oryzae* (Wang et al. 2018a). Here we showed that miR1432 coordinated rice yield and immunity via different target genes. Blocking miR1432 enhanced both yield and resistance accompanied by enhanced expression of two target genes playing different roles in the regulation of rice yield traits and resistance. *OsACOT* was high-expressed in the whole plant, whereas *OsEFH1* was only expressed in seedlings and almost not expressed in panicles (http://rice.plantbiology.msu.edu). Consistent with the spatial expression pattern, *OsEFH1* enhances resistance with a slight penalty in yield, whereas *OsACOT* improves rice yield. Therefore, the alteration of miR1432 can be exploited to improve rice yield and immunity simultaneously.

In mice, ACOT7 plays an important role in long-chain fatty acid elongation and counter-regulates fatty acid metabolism in neurons (Ellis et al. 2013). In rice, acyl-CoA thioesterases participate in the biosynthesis of medium-chain fatty acids and the regulation of lipid metabolism by hydrolyzing Acyl-CoA into free fatty acids and CoA. *OsACOT* was characterized as the 13th member of the acyl-CoA thioesterase superfamily (Zhao et al. 2019a; Ying et al. 2012). Overexpression of a miR1432-insensitive *OsACOT* leads to altered compositions of fatty acids, especially C:16 to C:18 in rice seeds, suggesting the role of *OsACOT* in the regulation of lipid and fatty acid metabolism (Zhao et al. 2019a). The metabolism of lipid and fatty acids is essential for endomembrane system organization, which played a key role in plant growth and development. For example, the Golgi, which belonged to the endomembrane system, was responsible for storage protein trafficking to the protein storage vacuole in rice endosperm cells (Liu et al. 2013). Conversely, the defects of endomembrane resulted in abnormal starch structure in rice endosperm cells and decreased grain filling (Wang et al. 2010). As a result, miR1432 manipulated rice grain filling by controlling the expression of *OsACOT*. However, the increase of grain weight of MIM1432 and OXACOT lines in our study was not as significant as that in the previous report (Zhao et al. 2019a). The possible reason was that the suppression on miR1432 offered by MIM1432 in our study was not as effective as that offered by STTM1432, and the *ACOT* overexpressed in OXACOT lines was not a mutant that could avoid the suppression by miR1432. As a result, although the MIM1432 and OXACOT lines showed increased yield, the increased levels in OXACOT lines were not as remarkable as those in the previous report. Moreover, MIM1432 exhibited significantly increased yield than OXACOT, suggesting that some other target genes of miR1432 also participated in the regulation of yield.

In this study, we showed that the miR1432-*OsEFH1* module regulates rice blast disease resistance and PTI responses. EFH family members contain the specific Ca^2+^-binding motif (helix–loop–helix structure, called EF-hand motifs), and act as Ca^2+^-sensor proteins to regulate the concentration of Ca^2+^ in plant cells (Zielinski 1998; Gifford et al. 2007). The Ca^2+^ influx in cells is a core event for PTI (Dodd et al. 2010; Boudsocq et al. 2010) and rice blast disease resistance (Wang et al. 2019). The recognition between PAMPs and plant receptors triggers a series of defense responses, including an increase of cytoplasmic calcium ([Ca^2+^]_cyt_), a burst of reactive oxygen species (ROS), and the expression of defense-related genes (Boller and He 2009). The elevation of [Ca^2+^]_cyt_ is implemented by Ca^2+^ influx from apoplast and intracellular stores (Thor and Peiter 2014). A recent study revealed that the ZAR1 resistosome is incorporated into planar lipid-bilayers and acted as a Ca^2+^ channel, and the activation of ZAR1 led to Glu11-dependent production of reactive oxygen species (ROS) (Bi et al. 2021). Blocking miR1432, or overexpression of *OsEFH1* enhances PTI responses, indicating that miR1432 regulates rice immunity by controlling PTI responses via *OsEFH1*. However, it is still unclear that whether *OsEFH1* directly binds Ca^2+^ and regulates Ca^2+^ influx. Moreover, overexpression of *OsEFH1* results in decreased grain number leading to reduced grain yield, indicating that *OsEFH1* plays a negative role in the regulation of rice yield by suppressing the development of panicles (Fig. 7c). Over 20 calcium-dependent protein kinase (CDPK) genes were up- or down-regulated in panicles during rice reproductive developmental stages (Ray et al. 2007), indicating the participation of Ca^2+^-binding proteins in panicle development. Growth-defense tradeoffs are thought to occur because of resource restrictions, and hormone crosstalk acted as a major player in regulating the balance of tradeoffs (Huot et al. 2014). Moreover, transcriptional reprogramming of the immunity-associated genes in plants often reduces plant growth and yield (Ha et al. 2021). Whether *OsEFH1* regulated rice panicle development via these CDPK or hormone signaling, or the transcriptional reprogramming is unknown and needs further study.

## Methods

### Plant Materials and Growth Conditions

The rice (*Oryza sativa* L.) accessions Lijiangxin Tuan Heigu (LTH), International Rice Blast Line Pyricularia-Kanto51-m-Tsuyuake (IRBLkm-Ts), and Nipponbare (ssp. *japonica*) were used in this study. For resistance assay, the rice plants were grown in a greenhouse with a 28/23 ± 1 °C day/night temperature, 70% relative humidity, and a light/dark period of 14 h/10 h. For yield traits assay, the rice plants were grown in a paddy field in Wenjiang District, Chengdu, China (36° N, 103° E) during the normal rice-growing season from mid-April to late-September.

### Plasmid Construction and Genetic Transformation

The transgenic lines were generated following a previous report (Li et al. 2017). To construct the transgenic lines overexpressing Osa-miR1432, the sequence of the *MIR1432* gene containing 213 bp upstream and 247 bp downstream sequences was amplified from NPB genomic DNA with primers Osa-miR1432-F and Osa-miR1432-R (Additional file 2: Table S2). We cloned the amplified fragment in binary vector 35S-pCAMBIA1300 and got the construct *p35S: MIR1432* overexpressing miR1432. To construct the target mimic of Osa-miR1432, the target mimic sequences of Osa-miR1432 (GTCGGTGTCATAGTCTCTCCTGAT) containing the cutting sites of restrictive enzymes were formed by annealing with primers MIM1432-*Bam*HI-F and MIM1432-*Bgl*II-R (Additional file 2: Table S2). Then the annealing double-strand fragment was inserted into the Arabidopsis *IPS1* gene to substitute the target site of miR399 at *BamH*I and *Bgl*II sites as described previously (Franco-Zorrilla et al. 2007; Li et al. 2017). We cloned the reconstructed *IPS1*-MIM1432 fragment into the binary vector pCAMBIA1300 and got the construct *p35S: MIM1432* overexpressing a mimic of miR1432. Then the vectors *p35S: MIR1432* and *p35S: MIM1432* were transformed into Nipponbare via Agrobacterium strain EHA105 respectively to acquire the transgenic lines OX1432 and MIM1432. The positive transgenic lines were screened with Hygromycin B.

### Trait Measurements

The agronomic traits were measured from five plants growing in the middle of three rows in the paddy yard, including rice height, panicle number per plant, grain number per panicle, seed setting rate, 1000-grain weight, and yield per plant. The seeds were harvested at the full-mature stage and dried in a 42 °C oven for 1 week. Then the dried seeds were used to detect the yield traits, including grain number per panicle, seed setting rate, and 1000-grain weight using an SC-A grain analysis system (Wanshen Ltd., Hangzhou, China). These data were analyzed by a one-way ANOVA followed by post hoc Tukey HSD analysis with significant differences (*P* < 0.05).

### RNA Extraction and Gene Expression Analyses

Reverse-transcription-quantitative polymerase chain reaction (RT-qPCR) analyses were carried out to examine the accumulation of miR1432 and the expression of indicated genes in rice plants. Total RNAs were extracted from rice leaves using TRIzol reagent (Thermo Fisher Scientific, Chengdu, China) following the manufacturer’s instructions. To detect the expression of the indicated genes, the first-strand cDNA was synthesized from 1 μg of total RNA using Primescript RT reagent Kit with gDNA Eraser (TaKaRa Biotechnology, Dalian, China) according to the manufacturer's instruction. RT-qPCR was performed using specific primers and SYBR Green mix (QuantiNova SYBR Green PCR Kit, QIGEN, Chengdu, China) with BIO-RAD C1000TM Thermal Cycler (Bio-Rad Inc, Chengdu, China). The rice ubiquitin (*UBQ*) gene was used as an internal reference to normalize the relative expression levels of genes. The accumulation of miR1432 was examined in T0 plants. To determine the amounts of miR1432, total RNA was reverse-transcribed using a miRNA-specific stem-loop RT primer (Additional file 2: Table S2) with the PrimeScript™ RT reagent Kit with gDNA Eraser (Takara Biotechnology, Dalian, China), and the RT product was subsequently used as a template for RT-qPCR by using miRNA-specific forward primers and the universal reverse primer (Additional file 2: Table S2). snRNA U6 was used as an internal reference to normalize the relative amounts of miR1432. RT-qPCR analyses were performed with three technical replicates. The 2^−∆∆CT^ method was exploited to analyze the relative expression levels of miRNAs.

#### Chitin Treatment

Three-leaf-stage LTH and IRBLKm-Ts seedlings were sprayed with 10 µg mL^−1^ chitin (Sigma, Merck Life Science (Shanghai) Co., Ltd., Shanghai, China), and the leaf samples were collected at 1, 3, and 6 hpi for RT-qPCR assay.

### Pathogen Infection and Microscopy Analysis

*Magnaporthe oryzae* strain Guy11, DZ96, RB22, 97-27-2, and the eGFP-tagged strain Zhong8-10-14 (GZ8) were used for blast-disease resistance and defense response assays. These *M*. *oryzae* strains were cultured in plates containing oat-tomato-agar (OTA) medium at 28 ℃ for 2 weeks with 12-h/12-h light/dark cycles. After getting rid of the surface mycelia with distilled water, the plates were further incubated for 3 days to promote sporulation. Then the spores were collected with distilled water and the concentration of which was diluted to 1 × 10^5^ or 5 × 10^5^ conidia mL^−1^ for inoculation.

For the invasive process assay, the diluted spores were inoculated on 5-cm-long leaf sheaths as described previously (Kankanala, 2007 #1048). Then the inoculated epidermal layer was excised and the invasive process, including conidia germination, appressorium development, and invasive hyphae growth, were recorded at 24 to 48 hpi by a Nikon A1 Laser Scanning Confocal Microscopy (Nikon Instruments, Inc., Shanghai, China). The quantitative analysis of the invasive process was conducted following the protocol described previously (Li, 2014 #1068).

For resistance assay, wound- or spray-inoculation was used following a previous report (Kong, 2012 #1469). Briefly, conidia suspension (5 × 10^5^ conidia mL^−1^) of indicated strains was wound-inoculated at the wounded sites or spray-inoculated on the three- to five-leaf-stage seedlings. Lesion formation was examined at 4–6 days post-inoculation. The fungal biomass was determined by using the DNA amounts of fungal *Mopot2* against rice DNA amounts of ubiquitin via RT-qPCR (Li et al. 2017).

### H_2_O_2_ Accumulation Assay

To observe the H_2_O_2_ accumulation in rice plants, three-leaf-stage seedlings were inoculated with *M*. *oryzae* strain Guy11 at the concentration of 5 × 10^5^ conidia mL^−1^. At 40 hpi, leaves were collected and incubated in 1 mg/mL DAB (Sigma, Merck Life Science Co., Ltd. Shanghai, China) at 22 °C for 8 h at illumination. The DAB-stained leaves were cleaned in 95% ethanol and then observed under a microscope (Zeiss imager A2, Carl Zeiss (Chengdu) Co. Ltd, China).

### Agrobacterium-Mediated Transient Expression Assay in *Nicotiana benthamiana*

YFP detection and accumulation were conducted following a previous report (Li, 2017 #1206). To generate *EFH1-YFP* reporter fusions, we fused *YFP* with the cDNA sequence of *OsEFH1* at its N-terminus. The fused fragments were inserted into *Kpn*I-*Spe*I sites of binary vector 35S-pCAMBIA1300 (*35S: OsEFH1-YFP*). Then the vector was transformed into Agrobacterium strain GV3101 for agroinfection assay in *N. benthamiana*. In brief, Agrobacterium strain GV3101 harboring the respective expression constructs (*35S: OsEFH1-YFP*, *35S: miR1432*, *35S: MIM1432*) was incubated at 28 °C overnight in liquid LB media containing antibiotics kanamycin (50 mg/mL) and carbenicillin (50 mg/mL) on a table shaking at 250 rpm. The Agrobacterium were collected and resuspended in an MMA buffer (10 mM MES,10 mM MgCl2, 100 mM AS) and infiltrated into leaves of *N. benthamiana* for transient expression assay. Leaves were examined at 48 hpi using a Nikon A1 Confocal Laser Scanning Microscope (Nikon Instruments, Inc., China). The detection of EFH1-YFP fused protein was assayed with BioRad Image soft. The relative protein mass was calculated as the ratio of the mass of *OsEFH1-YFP* to the mass of HSP.

### PTI-Related Defense Responses

The leaves of three to five-leaf-stage rice seedlings were selected to conduct the production of reactive oxygen species (ROS). The leaves of *N. Benthamiana* were used to transiently express miR1432 with or without MIM1432 and *OsEFH1*. The mRNA levels of miR1432, *MIM1432* and *OsEFH1* were analyzed as the methods in the “RNA extraction and gene expression analyses” section. For the ROS assay, the leaves were cut with a 5-mm-diameter hole punch and the punched circular leaves were cut into 1-mm-width and incubated in 200 µL water in a 96-well plate for 16 h. Then the leaves were treated with or without 1 µM flg22 or 20 µg/mL chitin in 200 µL buffer containing 20 mM L-012 (Wako, Japan), 10 µg/mL horseradish peroxidase (Sigma-Aldrich Shanghai Trading Co Ltd, Shanghai, China). The production of ROS was detected using a GLOMAX96 Microplate Luminometer (Promega Biotech Co., Ltd, Beijing, China) for 30–60 min and determined as relative luminescence units. We examine the PTI-triggered callose deposition in rice following a previous report (Liu et al. 2012). The rice was planted for 5 days and the first leaves were cut and treated with flg22 or chitin for 12 h. Then the treated leaves were fixed in ethanol: acetic acid (3:1 [v/v]) solution for 5 h and rehydrated in 70% and 50% ethanol for 2 h, respectively, and in water overnight. Then the decolored leaves were treated with 10% NaOH for 1 h to make the tissues transparent. The transparent leaves were washed three times with water and incubated in the staining buffer containing 150 mM K_2_HPO_4_, pH 9.5, 0.01% aniline blue (Sigma-Aldrich) for 4 h. We used a fluorescence microscope (Zeiss imager A2.0) to capture the images of callose deposition under a UV channel (340 to 380 nm) and calculated the callose deposits using Image J software.

## Supplementary Information

**Additional file 1: Figure S1.** Rice miR1432 is responsive to blast fungus. **a** The disease lesions on leaves of LTH and IRBLkm-Ts with the inoculation of *Magnaporthe oryzae* strain Guy11. The photo was captured five days post-inoculation. **b** The Reverse transcription-quantitative polymerase chain reaction (RT-qPCR) data show miR1432 levels in LTH and IRBLkm-Ts with or without Guy11 treatment. Data are shown as mean ± SD (n= 3 independent samples). Different letters above the bars show significant differences (*P* < 0.01) as determined by the One-way Tukey-Kramer test. These experiments were repeated two times with similar results. **Additional file 1: Figure S2.** miR1432 is predicted to target *OsEFH1* and *OsACOT*. The sequence alignment of miR1432, MIM1432, and the target sites of the predicted target genes. Mismatched nucleotides were highlighted in red colors. **Additional file 1: Figure S3.** miR1432 regulates rice resistance to *Magnaporthe oryzae*. **a** The disease phenotypes on leaves of MIM1432 and the Nipponbare control following spray-inoculation with *M. oryzae* strain Guy11. The phenotype was captured five days post-inoculation. Bar = 5 mm. **b** Quantification analysis of the fungal biomass in a. The relative fungal biomass was measured by using the ratio of DNA level of *M. oryzae MoPot2* genes against the rice genomic *ubiquitin* DNA level. The rice *ubiquitin* gene was used as the internal reference gene. **c** The mRNA levels of the defense-related genes in OX1432, MIM1432, and the Nipponbare control 12 hours post-inoculation. For b and c, error bars indicate SD (n = 3 independent samples). Different letters above the bars indicate a significant difference (*P* < 0.01) as determined by a one-way Tukey-Kramer analysis. Similar results were obtained in at least two independent experiments. **Additional file 1: Figure S4.** Overexpression of *OsEFH1* enhances rice blast disease resistance. **a** The reverse transcription-quantitative polymerase chain reaction (RT-qPCR) data show the mRNA levels of *OsEFH1* in LTH and IRBLkm-Ts with or without Guy11 treatment. **b** The disease phenotypes on leaves of OXEFH1 and the Nipponbare control following spray-inoculation of *M. oryzae* strain GZ8 five days post-inoculation. Bars = 5 mm. **c** Quantification analysis of the fungal biomass in b. The relative fungal biomass was measured by using the ratio of DNA level of *M. oryzae Pot2 * genes against the rice genomic *ubiquitin* DNA levels. **d** The invasion process of GZ8 at 24 hours post-inoculation (hpi) and the H_2_O_2_ accumulation at 48 hpi in the invasive sheath cells of OXEFH1 and the Nipponbare control. Bars = 40 µm. For a and c, data are shown as mean ± SD (n = 3 independent samples). Different letters above the bars show significant differences (*P* < 0.01) as determined by the One-way Tukey-Kramer test. Similar results were obtained in at least two independent experiments. **Additional file 1: Figure S5.** Chitin regulates the expression of *Pikm* genes in IRBLkm-Ts. The reverse transcription-quantitative polymerase chain reaction (RT-qPCR) data show the mRNA levels of *Pikm1-Ts* and *Pikm2-Ts* in IRBLkm-Ts with or without chitin treatment. Data are shown as mean ± SD (n= 3 independent samples). **Additional file 1: Figure S6.** The miR1432-OsEFH1 module regulates PAMPs-induced burst of reactive oxidative species (ROS). **a**–**c** The relative miR1432 levels, *MIM1432* levels, and mRNA levels of *OsEFH1* in the leaves transiently expressing *MIR1432* with or without *MIM1432* or *OsEFH1*, respectively. **d** and **e** The burst of ROS induced by flg22 (d) and chitin (e) in the leaves of *Nicotiana benthamiana* transiently expressing miR1432 with or without MIM1432 or *OsEFH1*, respectively. The leaves transiently expressing YFP are used as the control. Data are shown as mean ± SD (n = 4 independent repeats). Similar results were obtained in at least two independent experiments.

**Additional file 2: Table S1.** The agronomical traits of the indicated lines. **Table S2.** Primers used in this study.

## Data Availability

All the datasets are included within the article and its additional files.

## Abbreviations

LTH: Lijiangxin Tuan Heigu
IRBLkm-Ts: International Rice Blast Line Pyricularia-Kanto51-m-Tsuyuake
NPB: Nipponbare
CRISPR: Clustered regularly interspaced short palindromic repeats
OTA: Oat-tomato-agar
RT-qPCR: Reverse transcription quantitative polymerase chain reaction
LSCM: Laser scanning confocal microscopy
OsEFH1: EF-hand family protein 1
OsACOT: Acyl-CoA thioesterase
ROS: Reactive oxygen species
PAMP/MAMP: Pathogen/microbe-associated molecular pattern
PTI: PAMP/MAMP-triggered immunity
ETI: Effector-triggered immunity
PRRs: Pattern recognition receptors
HR: Hypersensitive response
miRNAs: MicroRNAs
*M. oryzae*: *Magnaporthe oryzae*
*DCL1*: *Dicer-like 1*
GZ8: GFP-tagged Zhong8-10-14
OX1432: The transgenic lines overexpressing miR1432
MIM1432: The transgenic lines overexpressing a target mimic of miR1432
OXEFH1: The transgenic lines overexpressing *OsEFH1*
OXACOT: The transgenic lines overexpressing *OsACOT*
*NAC4*: *NAC DOMAIN-CONTAINING PROTEIN 4*
*KS4*: *ENT-KAURENE SYNTHASE 4*
*PR1a*: *PATHOGENESIS-RELATED GENE 1a*
SSR: Seed setting rate
NPR1: Non-expressor of Pathogenesis-Related genes1
uORF: Upstream open reading frame
IPA1: Ideal Plant Architecture 1
WRKY: WRKYGQK domain
*DEP1*: *Dense and Erect Panicle 1*
snRNA: Small nuclear RNA
