# Supplementary material for: Rice miR1432 Fine-Tunes the Balance of Yield and Blast Disease Resistance via Different Modules
Source: Rice (N Y). 2021 Oct 21;14:87. doi: 10.1186/s12284-021-00529-1 (PMC8531185; doi:10.1186/s12284-021-00529-1)
Supplement: Supplementary file 1 — Additional file 1: Figure S1. Rice miR1432 is responsive to blast fungus. a The disease lesions on leaves of LTH and IRBLkm-Ts with the inoculation of Magnaporthe oryzae strain Guy11. The photo was captured five days post-inoculation. b The Reverse transcription-quantitative polymerase chain reaction (RT-qPCR) data show miR1432 levels in LTH and IRBLkm-Ts with or without Guy11 treatment. Data are shown as mean ± SD (n= 3 independent samples). Different letters above the bars show significant differences (P < 0.01) as determined by the One-way Tukey-Kramer test. These experiments were repeated two times with similar results. Additional file 1: Figure S2. miR1432 is predicted to target OsEFH1 and OsACOT. The sequence alignment of miR1432, MIM1432, and the target sites of the predicted target genes. Mismatched nucleotides were highlighted in red colors. Additional file 1: Figure S3. miR1432 regulates rice resistance to Magnaporthe oryzae. a The disease phenotypes on leaves of MIM1432 and the Nipponbare control following spray-inoculation with M. oryzae strain Guy11. The phenotype was captured five days post-inoculation. Bar = 5 mm. b Quantification analysis of the fungal biomass in a. The relative fungal biomass was measured by using the ratio of DNA level of M. oryzae MoPot2 genes against the rice genomic ubiquitin DNA level. The rice ubiquitin gene was used as the internal reference gene. c The mRNA levels of the defense-related genes in OX1432, MIM1432, and the Nipponbare control 12 hours post-inoculation. For b and c, error bars indicate SD (n = 3 independent samples). Different letters above the bars indicate a significant difference (P < 0.01) as determined by a one-way Tukey-Kramer analysis. Similar results were obtained in at least two independent experiments. Additional file 1: Figure S4. Overexpression of OsEFH1 enhances rice blast disease resistance. a The reverse transcription-quantitative polymerase chain reaction (RT-qPCR) data show the mRNA levels of OsE [file 12284_2021_529_MOESM1_ESM.pdf]

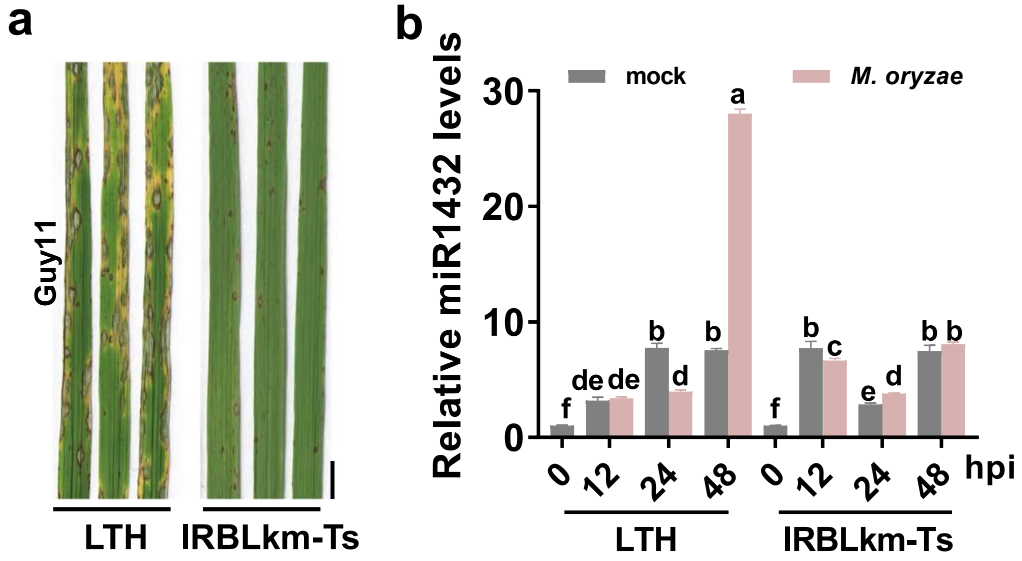

Additional file 1: Figure S1. Rice miR1432 is responsive to blast fungus. **a** The disease lesions on leaves of LTH and IRBLkm-Ts with the inoculation of *Magnaporthe oryzae* strain Guy11. The photo was captured five days post-inoculation. **b** The Reverse transcription-quantitative polymerase chain reaction (RT-qPCR) data show miR1432 levels in LTH and IRBLkm-Ts with or without Guy11 treatment. Data are shown as mean ± SD (n= 3 independent samples). Different letters above the bars show significant differences ($P < 0.01$) as determined by the One-way Tukey-Kramer test. These experiments were repeated two times with similar results.

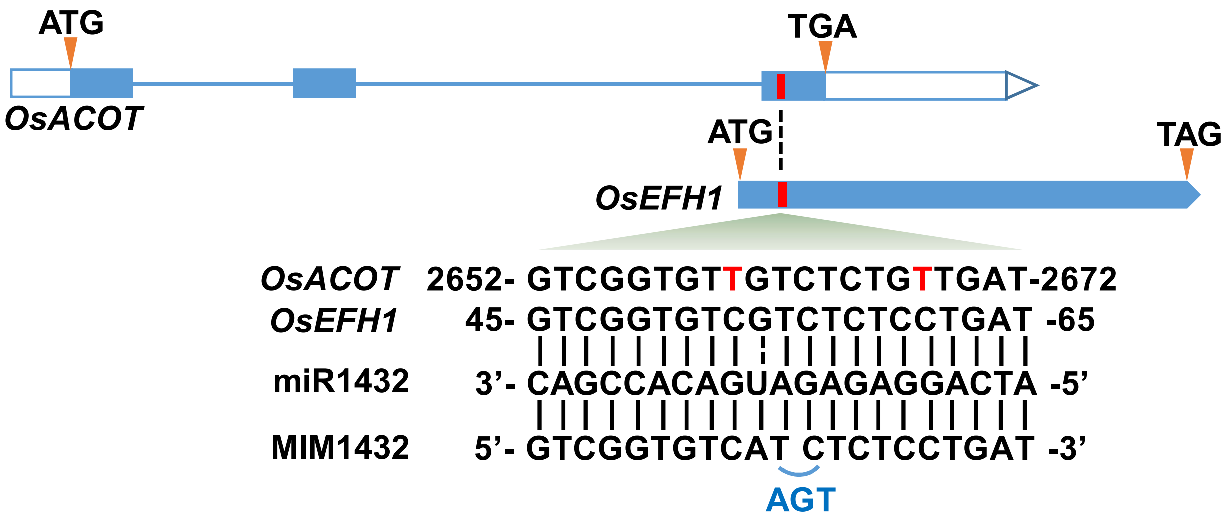

Additional file 2: Figure S2. miR1432 is predicted to target *OsEFH1* and *OsACOT*. The sequence alignment of miR1432, MIM1432, and the target sites of the predicted target genes. Mismatched nucleotides were highlighted in red colors.

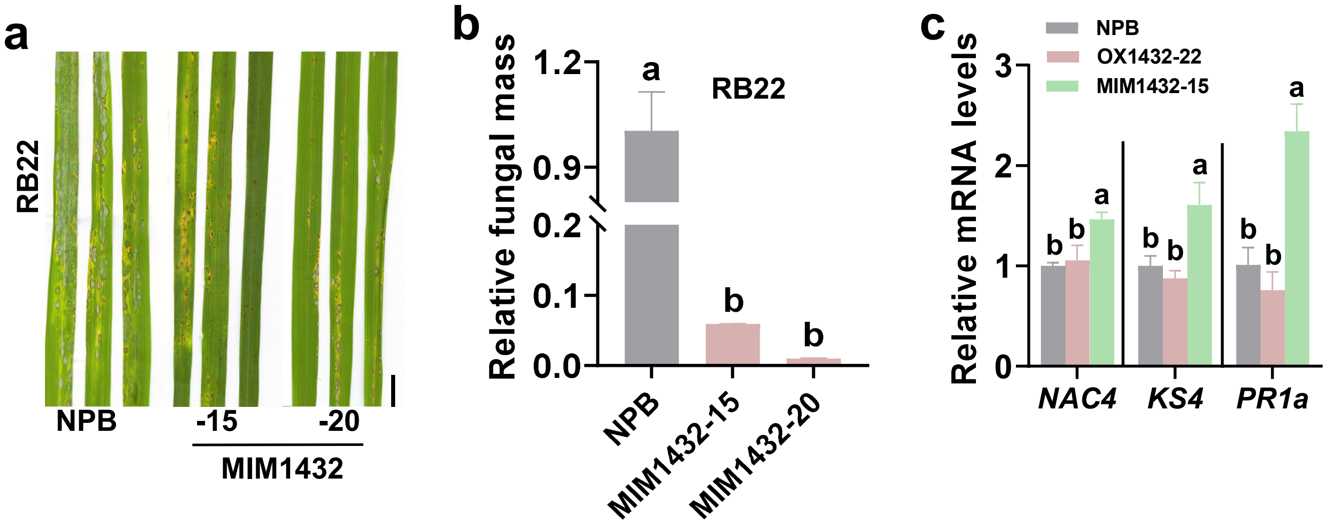

Additional file 3: Figure S3. miR1432 regulates rice resistance to *Magnaporthe oryzae*. **a** The disease phenotypes on leaves of MIM1432 and the Nipponbare control following spray-inoculation with *M. oryzae* strain Guy11. The phenotype was captured five days post-inoculation. Bar= 5 mm. **b** Quantification analysis of the fungal biomass in a. The relative fungal biomass was measured by using the ratio of DNA level of *M. oryzae MoPot2* genes against the rice genomic *ubiquitin* DNA level. The rice *ubiquitin* gene was used as the internal reference gene. **c** The mRNA levels of the defense-related genes in OX1432, MIM1432, and the Nipponbare control 12 hours post-inoculation. For b and c, error bars indicate SD (n = 3 independent samples). Different letters above the bars indicate a significant difference ($P < 0.01$) as determined by a one-way Tukey-Kramer analysis. Similar results were obtained in at least two independent experiments.

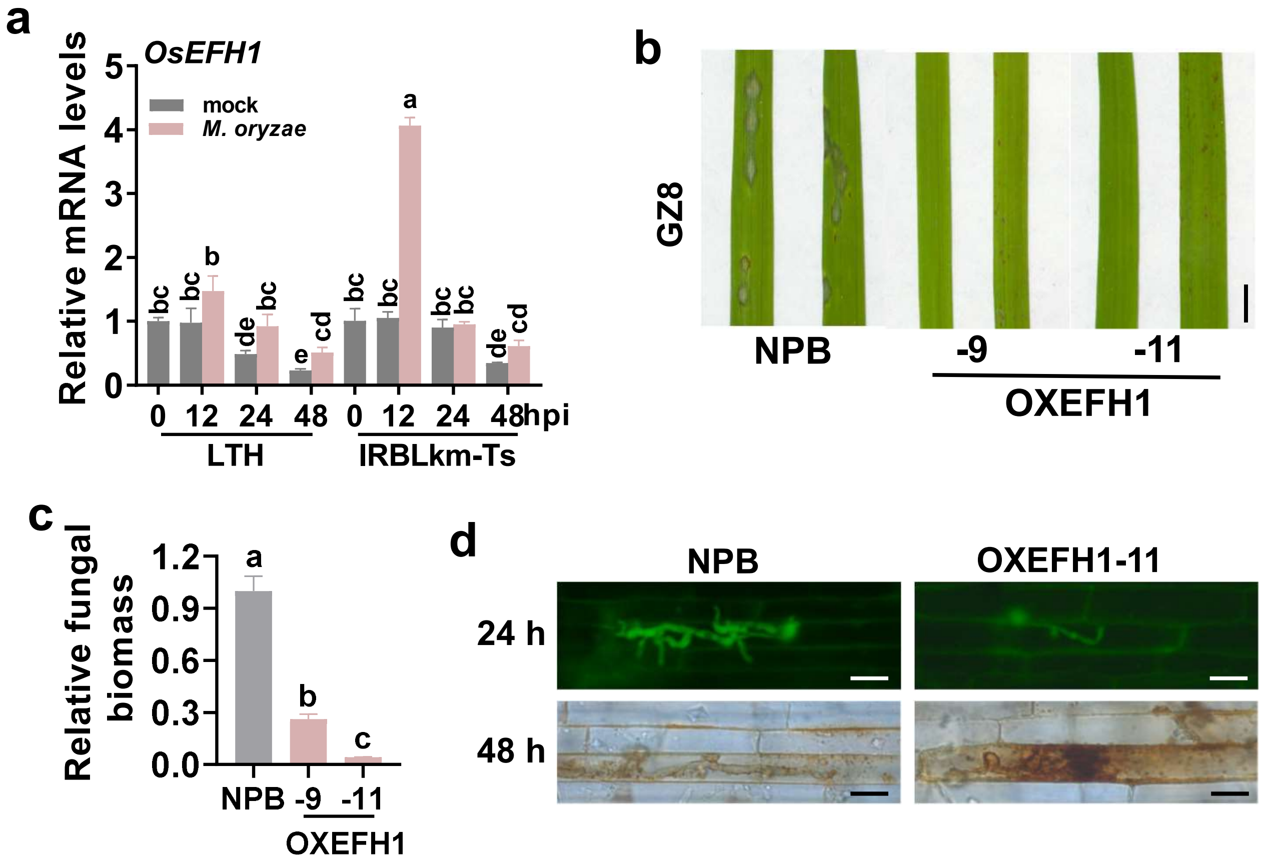

Additional file 4: Figure S4 Overexpression of *OsEFH1* enhances rice blast disease resistance. **a** The reverse transcription-quantitative polymerase chain reaction (RT-qPCR) data show the mRNA levels of *OsEFH1* in LTH and IRBLkm-Ts with or without Guy11 treatment. **b** The disease phenotypes on leaves of OXEFH1 and the Nipponbare control following spray-inoculation of *M. oryzae* strain GZ8 five days post-inoculation. Bars = 5 mm. **c** Quantification analysis of the fungal biomass in b. The relative fungal biomass was measured by using the ratio of DNA level of *M. oryzae Pot2* genes against the rice genomic *ubiquitin* DNA levels. **d** The invasion process of GZ8 at 24 hours post-inoculation (hpi) and the H_2O_2 accumulation at 48 hpi in the invasive sheath cells of OXEFH1 and the Nipponbare control. Bars= 40 µm. For a and c, data are shown as mean ± SD (n= 3 independent samples). Different letters above the bars show significant differences ($P < 0.01$) as determined by the One-way Tukey-Kramer test. Similar results were obtained in at least two independent experiments.

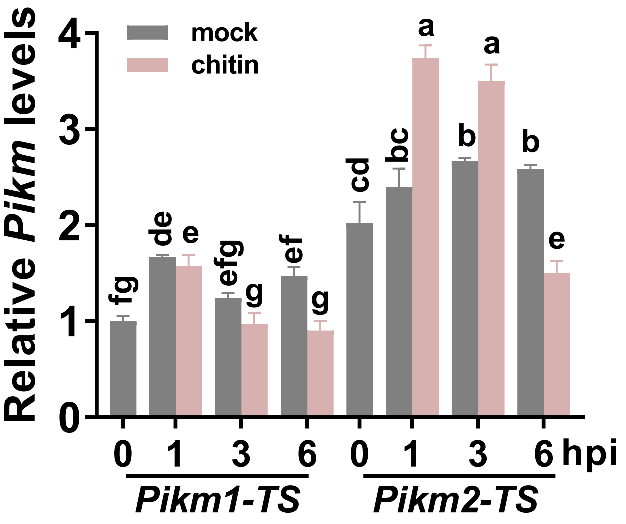

Additional file 5: Figure S5. Chitin regulates the expression of *Pikm* genes in IRBLkm-Ts. The reverse transcription-quantitative polymerase chain reaction (RT-qPCR) data show the mRNA levels of *Pikm1-Ts* and *Pikm2-Ts* in IRBLkm-Ts with or without chitin treatment. Data are shown as mean ± SD (n= 3 independent samples).

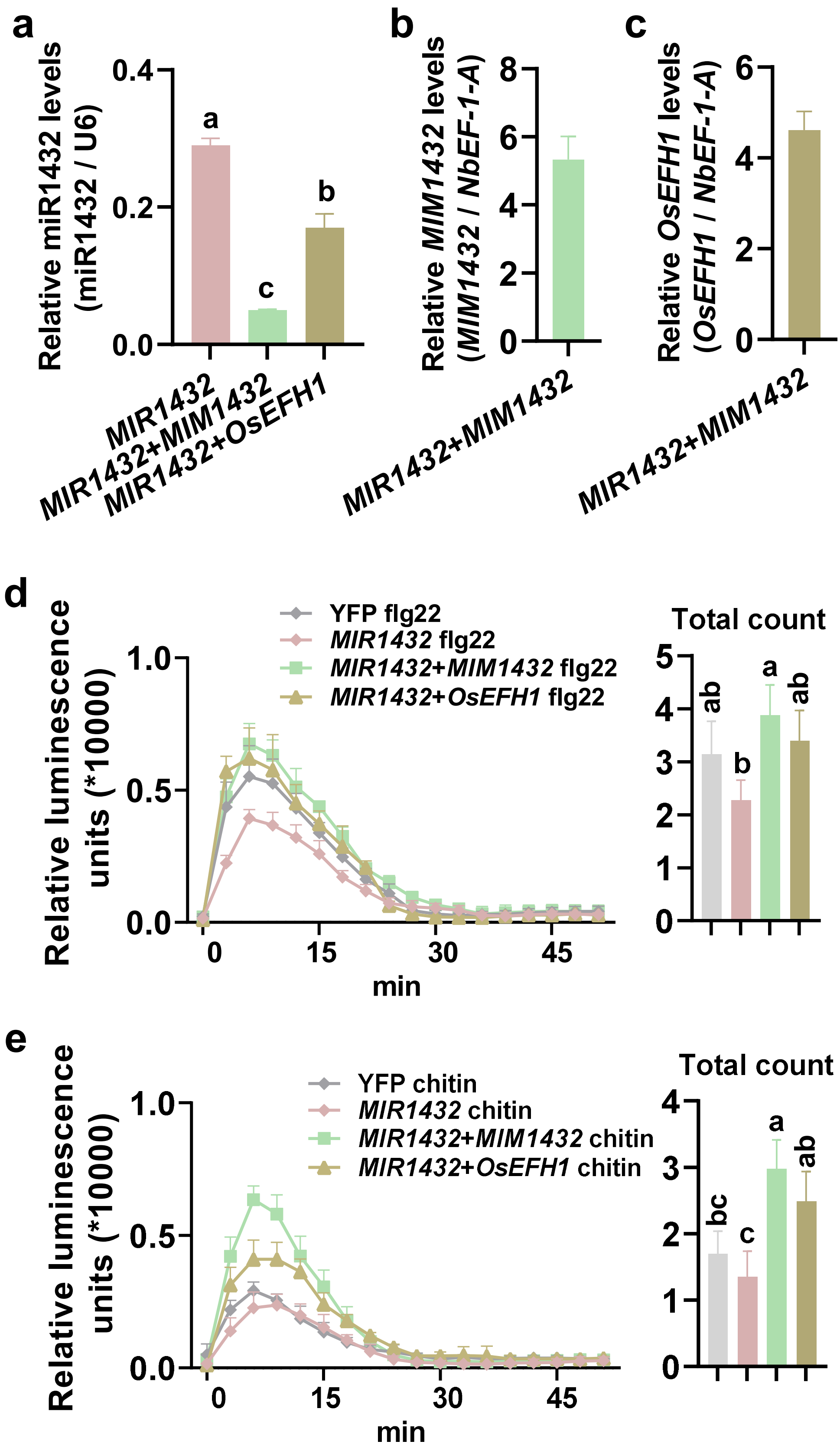

Additional file 6: Figure S6. The miR1432-*OsEFH1* module regulates PAMPs-induced burst of reactive oxidative species (ROS). **a-c** The relative miR1432 levels, *MIM1432* levels, and mRNA levels of *OsEFH1* in the leaves transiently expressing *MIR1432* with or without *MIM1432* or *OsEFH1*, respectively. **d** and **e** The burst of ROS induced by flg22 (d) and chitin (e) in the leaves of *Nicotiana benthamiana* transiently expressing miR1432 with or without MIM1432 or *OsEFH1*, respectively. The leaves transiently expressing YFP are used as the control. Data are shown as mean ± SD (n= 4 independent repeats). Similar results were obtained in at least two independent experiments.
